# The Scope of Telemedicine in Nepal during COVID-19 Pandemic

**DOI:** 10.31729/jnma.6692

**Published:** 2021-12-31

**Authors:** Sanjib Koirala, Bibek Raj Parajuli

**Affiliations:** 1 Nepal Medical College and Teaching Hospital, Jorpati, Kathmandu, Nepal; 2 NMVS Research Academy, Nepal Medical Volunteer Society, Morang, Nepal; 3 College of Medical Sciences, Bharatpur, Chitwan, Nepal

**Keywords:** *COVID-19*, *Nepal*, *pandemics*, *telemedicine*

## Abstract

The government of Nepal proposed to provide health services for both COVID-19 and non-COVID-19 infected patients through telemedicine. After the outbreak of the COVID-19 in Nepal, the number of people taking online health services, including counselling, increased by 70% as compared to last year in the Kathmandu valley. People being depressed staying alone in isolation and quarantine can get an opportunity to talk and share their problems with doctors through the means of telemedicine. Telemedicine has saved the time, effort and money of people living in remote areas.

## INTRODUCTION

Nepal is a developing country in South Asia with rising use of science and technology. As COVID-19 has crippled the whole world, Nepal has had its fair share of problems and challenges. Telemedicine has played an important role in managing COVID-19 in Nepal, both directly and indirectly. The term “Telemedicine" can be defined as the services provided by a physician via information and communication technologies (ICTs). “Telehealth" is a broader term that refers to all health-related services provided through ICTs which ranges from public health messages to services provided by nurses or other skilled health workers. Usually, these terms are used synonymously.^1^

## TELEMEDICINE IN NEPAL

In the health sector emergency response plan, the Nepal government proposed to provide health services for both COVID and non-COVID patients, as well as mass counselling through telemedicine.^2^ Likewise, the Ministry of Health and Population allocated a budget of Rs 5 million for telemedicine in each province in the fiscal year 2019-20, though it has not started yet.^3^ Further, the government launched telemedicine services in all seven states of Nepal. It published the telemedicine focal person's name from eight major hospitals in seven states. According to the government, patients can get necessary health consultation and counselling services by contacting the listed doctors via SMS, phone, email and other means of communication.^4^

After the outbreak of the COVID-19 in Nepal, the number of people taking online health services, including counselling, increased by 70% as compared to last year in the Kathmandu valley.^5^ As Nepal has been severely affected by the second wave of coronavirus resulting in a shortage of beds in hospitals, the healthcare sector has faced a massive demand for digital healthcare systems and telemedicine services.^5^ Besides the government, different private organisations are also involved in providing online health services to COVID-19 patients in Nepal in their own homes by registered healthcare personnel through different means of communication such as Viber, WhatsApp etc.^5^ Numerous mobile applications have also been developed to provide teleconsultations which include both free and paid services.^6^ Some organizations have started telemedicine targeting Nepalese people living abroad free of cost.^3^ Information regarding preventive measures from coronavirus has also been provided by the different telecommunication service providers of Nepal.

In the present context, telemedicine has become an effective means to consult with healthcare providers from the comfort of one's own home. People being depressed staying alone in isolation and quarantine can get an opportunity to talk and share their problems with doctors through the means of telemedicine. Suicidal cases due to the effect of COVID-19 can be decreased by using telemedicine services. Telemedicine has saved the time, effort and money of people living in remote areas. People suffering from chronic disease and needing long term regular to follow up and advice can benefit from this service.^7^ Telemedicine has increased the comfort in seeking consultation for sexual and reproductive health, including abortion, in remote areas by minimizing the communication barrier for girls and women.^8^

Despite its benefits, telemedicine can be a double-edged sword. There may be chances of people staying home with disease relying solely on telemedicine. The inperson check-up is necessary for various diseases and further investigation, diagnostic tests and intervention may need hospital visits. Checking vitals and doing physical examination by the doctor is not possible via telemedicine, although history can be taken.^9^ The COVID-19 patient having happy hypoxia can be taken as a good example in this present context.

## WAYS FORWARD

Telemedicine must be taken as a supplement and not as a replacement for traditional in-person healthcare. Social media and video-chatting used for telemedicine should be replaced by authentic telemedicine applications. The rising trend of telemedicine must be kept up even after the pandemic so that people can still get the benefits of online consultations. However, the primary focus should be provided to in-person health facilities as virtual medicine cannot replace in-person healthcare. A proper bandwidth internet connection must be set up in rural areas of the nation so that the people in rural areas get maximum benefits from telemedicine. In addition, proper national guidelines must be formulated for the supervision and functioning of telemedicine services in Nepal. Further, telemedicine services must be upgraded to the international platform which may benefit the patients worldwide.
